# Using Smart Home Technologies to Promote Physical Activity Among the General and Aging Populations: Scoping Review

**DOI:** 10.2196/41942

**Published:** 2023-05-12

**Authors:** Kiemute Oyibo, Kang Wang, Plinio Pelegrini Morita

**Affiliations:** 1 Department of Electrical Engineering & Computer Science York University Toronto, ON Canada; 2 School of Public Health Sciences University of Waterloo Waterloo, ON Canada; 3 Department of Systems Design Engineering University of Waterloo Waterloo, ON Canada; 4 Centre for Digital Therapeutics Techna Institute University Health Network Toronto, ON Canada; 5 Institute of Health Policy, Management, and Evaluation University of Toronto Toronto, ON Canada

**Keywords:** smart home, physical activity, aging population, activity of daily living, remote health care monitoring, health monitoring, health promotion, smart home technology, assisted living, mobile phone

## Abstract

**Background:**

Health-monitoring smart homes are becoming popular, with experts arguing that 9-to-5 health care services might soon become a thing of the past. However, no review has explored the landscape of smart home technologies that aim to promote physical activity and independent living among a wide range of age groups.

**Objective:**

This review aims to map published studies on smart home technologies aimed at promoting physical activity among the general and aging populations to unveil the state of the art, its potential, and the research gaps and opportunities.

**Methods:**

Articles were retrieved from 6 databases (PubMed, CINAHL, Scopus, IEEE Xplore, ACM Library, and Web of Science). The criteria for inclusion were that the articles must be user studies that dealt with smart home or Active Assisted Living technologies and physical activity, were written in English, and were published in peer-reviewed journals. In total, 3 researchers independently and collaboratively assessed the eligibility of the retrieved articles and elicited the relevant data and findings using tables and charts.

**Results:**

This review synthesized 20 articles that met the inclusion criteria, 70% (14/20) of which were conducted between 2018 and 2020. Three-quarters of the studies (15/20, 75%) were conducted in Western countries, with the United States accounting for 25% (5/20). Activities of daily living were the most studied (9/20, 45%), followed by physical activity (6/20, 30%), therapeutic exercise (4/20, 20%), and bodyweight exercise (1/20, 5%). K-nearest neighbor and naïve Bayes classifier were the most used machine learning algorithms for activity recognition, with at least 10% (2/20) of the studies using either algorithm. Ambient and wearable technologies were equally studied (8/20, 40% each), followed by robots (3/20, 15%). Activity recognition was the most common goal of the evaluated smart home technologies, with 55% (11/20) of the studies reporting it, followed by activity monitoring (7/20, 35%). Most studies (8/20, 40%) were conducted in a laboratory setting. Moreover, 25% (5/20) and 10% (2/20) were conducted in a home and hospital setting, respectively. Finally, 75% (15/20) had a positive outcome, 15% (3/20) had a mixed outcome, and 10% (2/20) had an indeterminate outcome.

**Conclusions:**

Our results suggest that smart home technologies, especially digital personal assistants, coaches, and robots, are effective in promoting physical activity among the young population. Although only few studies were identified among the older population, smart home technologies hold bright prospects in assisting and aiding older people to age in place and function independently, especially in Western countries, where there are shortages of long-term care workers. Hence, there is a need to do more work (eg, cross-cultural studies and randomized controlled trials) among the growing aging population on the effectiveness and acceptance of smart home technologies that aim to promote physical activity.

## Introduction

### Rationale

Health-monitoring smart homes are becoming more and more popular, with some experts arguing that 9-to-5 health care service delivery might soon become a thing of the past [1-3]. Smart homes include digital sensing technologies and communication devices that can seamlessly and intelligently communicate with one another and with the outside world via the internet and mobile networks [1,4]. Examples of smart home devices include ambient devices, cameras, wearable sensors, fall prediction systems, fall detection medical alert systems [5,6], smart fridges, smart vacuum cleaners, smart washing machines, smart dryers, smart doorbells, smart toothbrushes, and smart home gym equipment [4,7,8]. The “smartness” of these devices and systems used in smart homes relates to their automated functions, such as context awareness, voice activation, event detection, activity monitoring, and data-driven artificial intelligence processing features that can adapt to the conditions of the homes and lifestyles of the home occupant [4]. A key measure of a “good” smart home is the extent to which it improves the security, safety, comfort, health, and well-being of the user and how unobtrusively it achieves it [9-12].

It has been proposed that, in the near future, especially in Western countries, we will be able to live in smart homes that have health and well-being built into their design and structure. Hence, smart homes are becoming the building blocks of futuristic smart cities where resources and big data can be shared effectively and intelligently with the aid of the Internet of Things (IoT). With smart homes, personalized health care services (eg, web-based care and remote patient monitoring [13,14]) can be provided to individual residents based on their unique needs, situations, and conditions [1,3]. IoT, which smart homes are a part of, is the interconnection of devices equipped with sensors, cameras, microphones, or actuators that can collect information from the environment automatically via the internet, enabling the devices to transmit and receive big data from one location to another without the need for human interaction [15]. According to experts, there are many great expectations with regard to how smart technologies and IoT will transform our everyday lives, including homes, but they often seem far-fetched and far from reality [16]. Research shows that household income, technology progressiveness, and energy conservation habits are among the key drivers of the purchase of smart home technologies [17].

Apart from energy conservation and security [9,15,18], one other key area where smart homes are being used is in the detection, monitoring, and promotion of physical activity among the general and aging populations [19,20]. Physical activity is important to the daily life of the general population, particularly the aging population and those living with certain health conditions. For example, the aging population requires physical activity to stay healthy and live and function independently [3,21,22]. In particular, home exercise has the potential to reduce fall risk factors associated with aging [20]. During an emergency period, such as the COVID-19 pandemic confining billions to their homes, smart home technology can be a useful, promising, and collaborative tool for promoting and assessing the physical and mental health of the general population [23].

However, there is limited work on understanding the landscape and state of the art with regard to smart home technologies aimed at promoting physical activity. Unlike previous reviews that focused on older people, especially those with certain health conditions, our scoping review focuses on the general population including young, middle-aged, and older people. Moreover, while previous reviews focused mainly on medication management, mobility, and falls, our review focuses on physical activity including activities of daily living (ADLs) and exercise. For example, Facchinetti et al [24] conducted a systematic review of how smart home technologies can help older people manage their chronic conditions. The authors found that smart home technologies possessed great potential in the management of chronic diseases among older people with cognitive impairment and in increasing patient safety. Similarly, Liu et al [25] conducted a systematic review and meta-analysis to examine the effect of smart home technologies on older people with chronic conditions. The authors found that most of the study participants were satisfied with smart homes and that the technology had a positive effect on the physical functioning and depression of older people with chronic conditions. However, the effect of tele-exercise on cognitive functioning was unclear. Lussier et al [26] conducted a systematic review to examine the effectiveness of smart home sensor technologies for the early detection of mild cognitive impairment through the monitoring of everyday life activities. The authors found that the sample sizes in the reviewed studies were very limited, making it difficult to establish the reproducibility of the studies’ results. Sapci et al [14] conducted a systematic review to identify the advances in assistive and aging-in-place smart home technologies for older adults and determine the level of evidence for their effectiveness. The authors found that the use of ubiquitous in-home monitoring and smart technologies has the potential to enhance the independence of older people and their health outcomes. Finally, Liu et al [25] conducted a systematic review to determine the levels of technology readiness among older people and provide evidence of the effectiveness of smart home technologies in monitoring the health of older people with complex needs to support their aging in place. The authors found that the level of technology readiness for smart homes and health-monitoring smart home technologies was still low. Moreover, the authors found no evidence that these technologies helped address fall prevention and health-related quality of life.

Our review of current scholarly studies regarding smart home technologies promoting physical activity indicated that they are fragmented, hence our choice of a scoping review rather than a systematic review. A decade ago, Morris et al [19] demonstrated in a systematic review based on a qualitative assessment the availability of a wide range of smart home technologies that support older people in living well and independently. However, the authors concluded that evidence on the effectiveness of using smart home technologies to promote a healthy and active life is sparsely documented [27]. Hence, this scoping review sets out to map the landscape of physical activity–promoting smart home technologies among the young, middle-aged, and older adult populations; synthesize the findings; report on their effectiveness; and identify the gaps and opportunities for future research. This review will serve as a first step in conducting a full systematic review in the near future as more studies are published on smart home technologies aimed at promoting physical activity.

### Objectives

The objective of this scoping review is to summarize and synthesize published studies on smart home technologies for promoting physical activity among the general and aging populations. The general and aging populations represent the working-age population (aged 15-63 years) and the older adult population (aged ≥64 years), respectively. The working-age population comprises 2 groups: the young population (aged 15-47 years) and the middle-aged population (aged 48-63 years) [28]. Most of the existing studies have been focused on the older adult population [11,19]. Thus, we set out to answer the following research questions: (1) How have smart home technologies been used to promote physical activity among the general and aging populations? (2) How effective are they? (3) What research gaps need to be filled and what new research opportunities need to be explored?

## Methods

### Overview

This scoping review followed a 3-stage approach proposed by Tranfield et al [29]. The stages include planning the review; conducting the review by analyzing selected articles; and reporting the elicited themes, findings, and recommendations. The planning stage included a preliminary scoping of the literature. This stage, undertaken by the first and third authors of the scoping review (KO and PPM), was aimed at identifying and refining the objectives of the review and developing a protocol. The protocol includes the search criteria and string for retrieving the articles from the databases (Multimedia Appendix 1), the selection criteria for including articles in the review, and the method of conducting the analysis of the included articles. The review conduction stage involved the systematic search of 6 databases by 3 researchers (KO, KW, and PM) using the search string developed in the first stage, screening the articles, selecting the final included articles, and analyzing them based on key themes. The 3 researchers searched 2 databases each. Whereas the first 2 researchers were the first and second authors of the scoping review, the third researcher was a research assistant. The reporting stage of the review process entailed reporting the descriptive statistics of the themes elicited from the included studies, the findings of the analysis undertaken, and the development of recommendations for future research [30].

### Eligibility Criteria

The inclusion and exclusion criteria are shown in Textbox 1. For example, the inclusion criteria were that the article must be a peer-reviewed journal paper and written in English. Moreover, the evaluated smart home or Active Assisted Living (AAL) technology must be evaluated with users (ie, participants). In the context of the scoping review, “users” refers to everyday people living in their individual homes, community residential dwellings, or nursing homes (eg, older adults and middle-aged people) at whom smart home technological interventions for promoting physical activity are targeted. In other words, users are noncaregivers and nonproviders of health care services. Finally, the study must comprise 2 aspects: “smart home” or “AAL” on the one hand and “physical activity” or “exercise” on the other hand, as seen in the search string in the next subsection.

**Eligibility criteria for including and excluding articles in the scoping review.**

**Inclusion criteria**

- Peer-reviewed journal articles
- Articles written in English
- Studies using primary data
- Studies evaluating a smart home intervention using participants
- Study participants comprising young, middle-aged, and older adult populations
- Study participants including people living at home, nursing homes, and community residential dwellings
- Studies conducted in laboratory or real-life settings
- Articles focusing on the 2 aspects of the review: smart home or Active Assisted Living and physical activity or exercise

**Exclusion criteria**

- Conference papers, book chapters, magazines, and web-based articles
- Articles not written in English
- Studies using secondary or open data sets
- Studies not evaluating a smart home intervention using participants
- Study participants aged <15 years
- Study participants including caregivers, physicians, nurses, and other health care providers
- Studies based on literature reviews
- Articles focusing on either aspect (smart home or Active Assisted Living or physical activity or exercise)

### Information Sources and Search Strategy

In total, 6 databases (PubMed, CINAHL, Scopus, IEEE Xplore, ACM Digital Library, and Web of Science) were searched between May 24, 2021, and June 1, 2021. The set of keywords used for the search included the following: (“smart home” OR smarthome OR “Active Assisted Living” OR “Ambient Assisted Living”) AND (“exercise” OR “fitness” OR “physical activity” OR “activity monitor”). We draw a distinction between “physical activity” and “exercise,” which are the focus of the scoping review as included in the search string. *Physical activity* is a broader term encompassing different ADLs, including exercise, dressing, chores, and ambulating, whereas *exercise* is a more specific term encompassing a structured, planned physical activity for the purpose of fitness or functional gains.

The retrieved articles were based on titles, abstracts, and keywords. Using the Mendeley Web Importer (Mendeley Ltd) integrated into the Google Chrome web browser, we exported the articles to a collaborative Mendeley reference system hosted in the cloud. Next, duplicates were removed from the retrieved articles, and the remaining articles were transferred to our collaborative Google spreadsheets for further screening.

### Selection of Sources of Evidence

The PRISMA-ScR (Preferred Reporting Items for Systematic Reviews and Meta-Analyses extension for Scoping Reviews) flowchart [31] was used to screen the articles retrieved from the databases and arrive at the final set of included articles. Screening was performed by 3 researchers (KW, PM, and HS), each of whom was assigned an approximately equal number of articles. The first researcher was the second author of the scoping review, whereas the second and third were research assistants. Screening began by removing duplicate articles from all the articles retrieved from the 6 databases, followed by eligibility determination, which entailed excluding ineligible articles based on their title or abstract. Finally, through a full-text review, ineligible articles were removed to arrive at the final set of included articles for the final analysis. During screening, articles that one or more screeners were not sure of (ie, whether they should be included in the next stage or not) were buffered and later discussed and resolved by the first 2 authors (KO and KW).

### Data Charting Process and Items

Textbox 2 shows the key data items that were elicited from each included article and their definition or description. The data items included author identification, study year, and outcome. For example, study outcome indicates whether the smart home technology evaluated by a given study was effective. In other words, was the smart home technology effective in detecting, classifying, recognizing, or promoting physical activity? If yes (eg, it increased physical activity), then the study was regarded as “positive.” If no (eg, it decreased physical activity), the study was regarded as “negative.” If no (eg, it did not have a substantial effect on exercise), the study was regarded as “no effect.” Moreover, if the smart home technology had a positive effect on some participants and a negative effect on others, it was regarded as “mixed.” Otherwise, it was regarded as “indeterminate” if we could not tell from the results and findings presented in the article. We used tables and bar graphs in Google spreadsheets to chart the data elicited from the included articles. For example, we presented all the machine learning algorithms used by the interventions in the included studies in a table as well as the various authors and the number and percentage of studies associated with each algorithm. Similarly, we presented all the interactive technologies evaluated in the included studies in a table as well as the various authors and the number and percentage of studies that evaluated each technology. Moreover, in the bar graphs, we presented a count of the respective values associated with each data item, such as study type, number of studies carried out in a given country, and number of articles published in a given year. The PRISMA-ScR checklist in Multimedia Appendix 2 [31] was used in reporting the results of the analysis and writing the scoping review.

**Study characteristics and descriptions.**

**Identification**

- Name of authors

**Study year**

- Year of publication

**Study type**

- Quantitative, qualitative, and mixed

**Country of study**

- Name of country in which the study was carried out

**Study month and year**

- The month and year in which the study was conducted

**Study duration**

- How long the study took (ie, the study period)

**Activity**

- The type of physical activity the study focused on

**Setting**

- Home, hospital, nursing home, or senior residential home

**Sensor type**

- Ambient, wearable, hybrid, or robot

**Interactive technology**

- The interactive technology evaluated by the study (eg, mobile app or web application)

**Participants**

- Participant profile, including sample size, gender, age, primary problem, and health status

**Intervention**

- The evaluated system for promoting physical activity

**Comparison**

- The alternative against which the intervention was compared

**Study outcome**

- The result of the intervention, which could be positive, negative, no effect, mixed, or indeterminate

**Findings or takeaways**

- Summary of the main findings and takeaways

## Results

In this section, we present the results of the article screening and selection process, the characteristics of the individual sources of evidence, and the charts and tables that address the 3 research questions.

### Selection of Sources of Evidence

**Figure 1:**
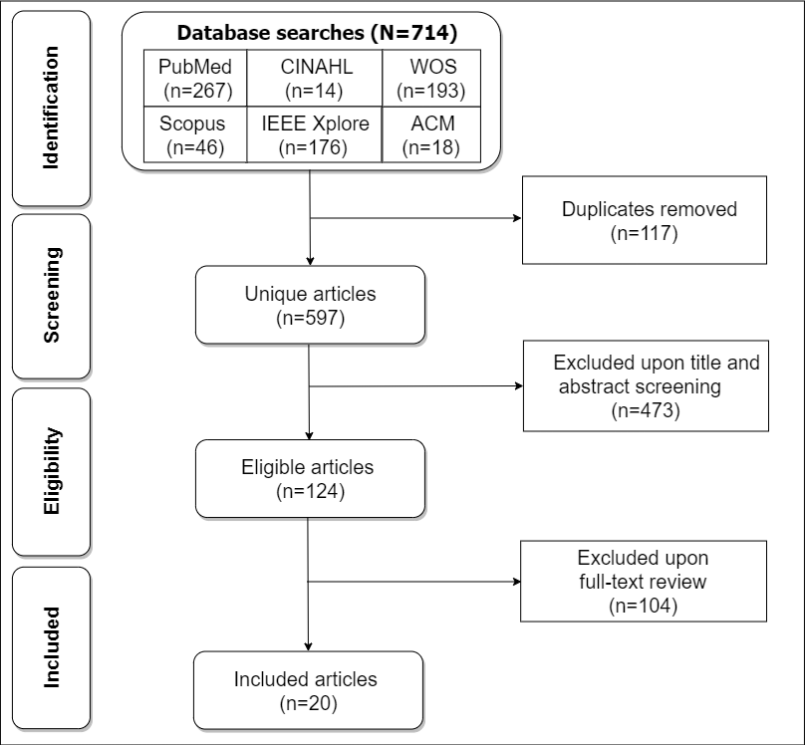
PRISMA (Preferred Reporting Items for Systematic Reviews and Meta-Analyses) flowchart for the screening and inclusion of articles in the scoping review. WOS: Web of Science.

Figure 1 shows the PRISMA-ScR flowchart, our search strategy for identifying, screening, and including eligible articles in the scoping review [31]. Overall, 714 articles were retrieved from the 6 databases that we searched. After removing 16.4% (117/714) of duplicates, we arrived at 597 unique articles. Next, we excluded 79.2% (473/597) of the articles that were found to be unrelated to the topic during the title and abstract screening. Finally, upon a full-text review of the remaining 124 articles, we excluded another 104 (83.9%) ineligible articles to arrive at 20 (16.1%) unique articles for the final data analysis.

### Characteristics and Results of Individual Sources of Evidence

The key characteristics described in Textbox 2 are presented in thematic tables and bar graphs and are described in the text. Table 1 shows a summary of the key information and the main findings for all 20 articles included in the scoping review. The tabulated information included the country in which each study was carried out; the duration of the study; and participant, intervention, comparison, and outcome information.

**Table 1:** Participant, intervention, comparison, outcome information, and summary of main findings of the included studies (N=20).

Study (country)	Type of study	Study month and year (duration)	Activity	Patient	Intervention	Comparison	Outcome	Summary of findings
Brauner and Ziefle [32], (Germany)	QN^a^	Not specified (not specified)	Exercise	Study 1: 71 participants, 35 male and 36 female; aged 20-86 years; mean age 48.4 years. Study 2: 64 participants, 32 male and 32 female; aged 17-85 years; mean age 43.2 years	A motion-based exercise game	N/A^b^	There was a decrease in pain, especially among older adults, after playing the game.	The study found that (1) serious games can be integrated into future AAL^c^ environments with invisibly integrated sensors and actuators supporting user interaction and (2) user diversity is essential when designing technology for older adults or people in need of care.
Chung et al [33], (South Korea)	QN	June 2018-July 2018 (3 months)	Rehabilitation exercise	16 participants (healthy), 5 male and 11 female; mean age 26.3 (SD 4.3) years	A developed wearable device	With and without motion artifact cancellation	Motion artifact cancellation provides more accurate heart rate estimation for all the exercise stages.	The study showed that a patient-provider interaction system can be an efficient cardiac rehabilitation exercise tool for remotely sharing cardiac rehabilitation exercise prescription and exercise records between patients and health care providers.
Görer et al [34], (Turkey)	MX^d^	Not specified (studies 1 and 2: 1 session per day, 10 min per session; study 3: 5 days, 1 day per week, 10-min daily session)	Exercise	Study 1: 9 participants, 3 female and 6 male; aged 25-35 years. Study 2: 6 participants, 5 female and 1 male; aged 70-80 years. Study 3: 12 female participants; aged 70-88 years	An autonomous exercise coach robot	N/A	Success level of engagement and motion of exercise under the instruction of coach robot	The study revealed that older people can successfully exercise with the assistance of a robot while staying engaged with the system over multiple sessions.
Gorjani et al [35], (Czech Republic)	QN	July 2019 (4 hours in each of 2 days)	ADL^e^	1 participant	A Konnex device and 2 wearable gadgets	N/A	Achieved an average human activity classification accuracy of 97.8%	The article proposed recognizing human activities in a single-occupant room using room air quality data (humidity, carbon dioxide, and temperature) in combination with movement-based data (accelerometer, gyroscope, and magnetometer). Given its promising outcome and highly accurate results, the technology holds potential in increasing the number of recognizable activity classes and making it possible to recognize activities within multiple-occupant rooms.
Helal et al [3], (United States)	QN	Not specified (not specified)	ADL	Studies 1 and 2: 20 participants each	A smart home–based health platform to recognize human activities	N/A	Achieved 98% recognition accuracy	The article proposed a flexible, extensible, and transparent smart home health platform that supports plug-and-play operation of new devices and components. It provides remote monitoring of patients with diabetes, including their activities, diet, and exercise compliance. It also helps evaluate the effects of alternative medicine and behavior regimens of patients with diabetes. The study used machine learning algorithms to analyze collected data on each patient and build a model of patient diet, exercise, activity, and health profile.
Dobbins et al [36], (United Kingdom)	QN	Not specified (not specified)	Physical activity	Data set 1: not specified; data set 2: 15 participants	Triaxial accelerometers and a heart rate monitor	N/A	Improvement on detecting physical activity with accuracies up to 99% and sensitivities of 100%	The study evaluated the performance of supervised machine learning in distinguishing physical activity. The evaluated classifier showed an improvement on existing studies, with accuracies of up to 99% and sensitivities of 100%.
Fung et al [37], (United States)	QN	Not specified (24 trials, did not specify how long a trial was)	Dynamic weight-shifting balance exercises	10 participants with Parkinson disease, 5 male and 5 female; mean age 70.7 years	An SBS^f^	N/A	The system substantially increased participants’ limits of stability in both anterior-posterior and medial-lateral directions.	The study focused on the usability and validation of an SBS. Participants were able to follow the target movements during an unsupervised session of dynamic weight-shifting balance exercises with the SBS in a laboratory setting and made improvements in their range of motion. The multimodal biofeedback of the SBS can provide movement guidance information to users as they perform the exercise session.
Biagetti et al [38], (Italy)	QN	Not specified (not specified)	Weight lifting exercise	3 participants	A classifier based on a wireless sEMG^g^ system	N/A	Achieved an overall accuracy of 85.7% by combining features extracted from acceleration and sEMG signals	The system consisted of several ultralight wireless sensing nodes that can acquire, process, and efficiently transmit the motion-related body signals to one or more base stations. The base stations were connected to a user interface software for viewing, recording, and analyzing the data. The system was tested on 4 different exercises and produced an overall accuracy of 85.7%.
Li et al [39], (United Kingdom)	QN	Not specified (not specified)	Physical activity	3 participants (all male); aged 26, 27, and 38 years	A passive Doppler radar	N/A	Achieved accuracy of 85%	Using passive radar as a noncontact sensor for breathing detection and activity recognition for health care applications. The experimental results showed that the proposed system provided adequate performance for both purposes and proved that a noncontact passive Doppler radar is a complementary technology to meet the challenges of future health care applications.
Costa et al [40], (Spain)	MX	Not specified (not specified)	Physical exercise	7 participants	A PHAROS^h^	N/A	Achieved accuracy of 97.35%	This article presented an interactive robot system designed for assisting older adults in their daily physical activities at home. The experimental results showed a high accuracy in detecting and classifying physical exercises.
Paay et al [41], (Denmark)	MX	Not specified (35 min on average per session)	Physical exercise	48 participants, 26 male and 22 female; mean age 28.3 years; aged 17-56 years	A digital personal assistant	With and without feedback from a digital personal assistant	Mixed, but had the potential to persuade people to increase physical activity indoors	There were significant differences regarding the number of repetitions participants performed and the time they spent exercising among the different persuasive test conditions. There was also a main effect of persuasive feedback mode on average heart rate. Moreover, the qualitative results showed that all 3 persuasive feedback modes persuaded participants to continue exercising and put effort into it. However, this depended on individual preferences for training approaches, tone of voice, and exercise experience level.
Rahman and Hossain [42], (Saudi Arabia)	QN	Not specified (not specified)	Physical activity	Not specified	A multi-sensor framework	N/A	Indeterminate; however, the framework holds potential in the detection of bodily motions	The proposed framework can provide live or statistical kinematic data, including rotational and angular range of motion of the joints of interest, and ambient environmental data, which can be shared with therapists and caregivers. Results showed that the proposed m-Therapy monitoring system can be deployed in real-life scenarios.
Sahu et al [43], (Canada)	QN	Not specified (1 week)	Physical activity	8 participants	Ecobee sensors	N/A	A strong association between sensor activation and the number of steps was observed.	The results showed a positive relationship between the total number of sensors activated and the total number of indoor steps traveled by study participants. Moreover, the indicators of sleep, physical activity, and sedentary behavior were all found to be highly comparable with those attained by the Public Health Agency of Canada.
Schmied et al [44], (Austria)	QL^i^	First round: May 2019; second round: November 2019 (436 minutes)	Evaluating participants’ perspective of a digital coach	First round: 17 participants, 7 male and 10 female; second round: 11 participants, 7 male and 4 female	A digital health coach	N/A	Indeterminate	The study found that the target group envisioned not only a coach for physical activity but a holistic coach that supported psychosocial well-being as well. In total, 4 functional requirements (physical activity and cognitive, emotional, and social support) were identified by the study.
Syed et al [45], (Saudi Arabia)	QN	Not specified (not specified)	Physical activity	10 participants	A smart home health care framework with multiple wearable sensors	N/A	Achieved overall accuracy of 97.1%	The proposed framework predicted 12 physical activities with an overall accuracy of 97.1%. This can be considered an optimal solution for recognizing physical activity to remotely monitor health conditions of older people.
Totty and Wade [46], (United States)	QN	Not specified (not specified)	Physical activity	10 participants, 4 male and 6 female; mean age 23.4 years	A gesture recognition sensor called Myo armband	N/A	Achieved accuracy of 89.2%	The study demonstrated that a KNN^j^ classifier, which combines sensor modalities (mean acceleration, mean angular rate of change, and sEMG data), can predict or classify ADLs with high accuracy (89.2%).
Cahill et al [47], (Ireland)	QL	Not specified (study 1: 10 half days; study 2: not specified; study 3: not specified)	ADL	Study 1: 104 participants; study 2: not specified; study 3: 22 participants	N/A	N/A	Indeterminate	This study identified the requirements for adapting new technology to enable resident wellness and person-centered care delivery in a residential care environment. Results indicated that independence and quality of life for older adults are linked to technology that enables interdependence and social communication among residents, caregivers, and family members.
Guan et al [48], (China)	QN	Not specified (not specified)	Physical activity	8 participants; height: 164-180 cm; weight: 45-74 kg	An infrared motion sensing system	N/A	Achieved classification rate of 97.71%	Experiment results showed that the proposed system (low measurement dimension, 1 module on the ceiling, and 2 modules on opposite tripods facing each other) can work well for classifying a variety of human activities.
Naranjo-Hernández et al [49], (Spain)	QN	Not specified (not specified)	ADL	6 participants, 3 male and 3 female; mean age 27.8 years	A wearable and low-cost smart accelerometer sensor for monitoring ADLs	N/A	Sensor recognized a variety of human activities	The system was able to (1) classify the level of activity that allows for the establishment of intensity and patterns of behavior, (2) distinguish activities such as climbing and descending stairs, and (3) estimate metabolic expenditure of the user independent of the activity performed and the user’s anthropometric characteristics.
Skubic et al [50], (United States)	QN	October 2005 (approximately 3 years)	ADL	34 participants with chronic illnesses; aged 70-95 years	A passive sensor network composed of passive infrared motion sensors	N/A	Able to monitor typical daily activity patterns	The sensors proved to be practical for the application. They could monitor daily activity patterns using the sensor network and recognize fall and spine movement of treadmill exercise.

^a^QN: quantitative study.

^b^N/A: not applicable.

^c^AAL: Active Assisted Living.

^d^MX: mixed study.

^e^ADL: activity of daily living.

^f^SBS: smart balance system.

^g^sEMG: surface electromyography.

^h^PHAROS: physical assistant robot system.

^i^QL: qualitative study.

^j^KNN: k-nearest neighbor.

### Synthesis of Results

#### Overview

**Figure 2:**
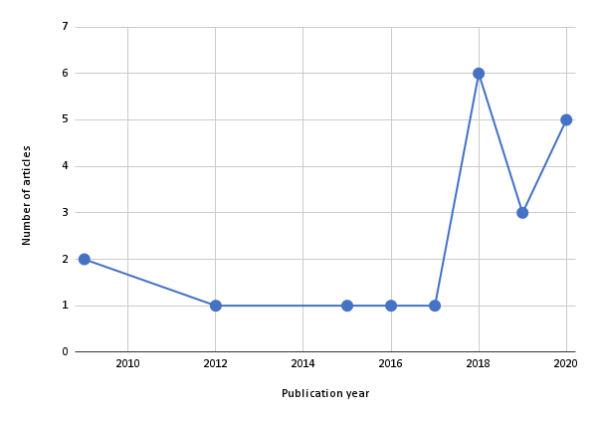
Distribution of the reviewed articles over a 12-year period.

In this section, we present the synthesized results of the analysis of the data elicited from the included articles. Figure 2 shows the number of articles published in a given year and the trend. Tables 2-5 present information that helps to address the research questions, such as how smart home technologies have been combined with artificial intelligence to promote physical activity among the general and aging populations, the types of technology, and their goals. Also presented in this section are the types of sensors, types of activities studied, study settings, and study outcomes.

**Table 2:** Machine learning algorithms used in the reviewed studies (N=20).

Algorithm	Reference	Studies, n (%)
KNN^a^	Totty and Wade [46] Guan et al [48]	2 (10)
NBC^b^	Syed et al [45] Guan et al [48]	2 (10)
SVM^c^	Li et al [39] Guan et al [48]	2 (10)
ANN^d^	Gorjani et al [35]	1 (5)
LR^e^	Gorjani et al [35]	1 (5)
CNN^f^	Costa et al [40]	1 (5)
RNN^g^	Costa et al [40]	1 (5)
GM-HMM^h^	Guan et al [48]	1 (5)
HMM^i^	Helal et al [3]	1 (5)
Not reported	Demiris et al [51]	1 (5)
N/A^j^	Brauner and Ziefle [32] Chung et al [33] Görer et al [34] Fung et al [37] Biagetti et al [38] Li et al [39] Paay et al [41] Rahman and Hossain [42] Sahu et al [43] Schmied et al [44] Cahill et al [47] Naranjo-Hernández [49]	12 (60)

^a^KNN: k-nearest neighbor.

^b^NBC: naïve Bayes classifier.

^c^SVM: support vector machine.

^d^ANN: artificial neural network.

^e^LR: logistic regression.

^f^CNN: convolutional neural network.

^g^RNN: recurrent neural network.

^h^GM-HMM: Gaussian mixture hidden Markov model.

^i^HMM: hidden Markov model.

^j^N/A: not applicable.

**Table 3:** Overall goals of the smart home technologies evaluated by the reviewed studies^a^ (N=20).

Goal	Reference	Studies, n (%)
Activity recognition	Helal et al [3] Gorjani et al [35] Dobbins et al [36] Biagetti et al [38] Li et al [39] Costa et al [40] Rahman and Hossain [42] Syed et al [45] Totty and Wade [46] Guan et al [48] Naranjo-Hernández et al [49]	11 (55)
Activity monitoring	Chung et al [33] Görer et al [34] Biagetti et al [38] Costa et al [40] Syed et al [45] Cahill et al [47] Demiris et al [51]	7 (35)
Biofeedback	Görer et al [34] Fung et al [37] Paay et al [41]	3 (15)
Exercise recommendation	Costa et al [40] Yoh et al [52]	2 (10)
Exercise demonstration	Görer et al [34] Sahu et al [43]	2 (10)
Exercise learning	Görer et al [34] Costa et al [40]	2 (10)
Persuasion	Brauner and Ziefle [32] Paay et al [41]	2 (10)
Coaching	Schmied et al [44]	1 (5)

^a^Activity in the table represents or is associated with physical activity or activities of daily living.

**Table 4:** Interactive technologies evaluated by the reviewed studies (N=20).

Technology	Reference	Studies, n (%)
Desktop application	Helal et al [3] Brauner and Ziefle [32] Biagetti et al [38] Costa et al [40] Schmied et al [44]	5 (25)
Web application	Chung et al [33] Costa et al [40] Rahman and Hossain [42] Cahill et al [47]	4 (20)
Mobile app	Chung et al [33] Fung et al [37] Syed et al [45] Cahill et al [47]	4 (20)
Robot	Görer et al [34] Costa et al [40] Schmied et al [44]	3 (15)
Smartwatch app	Dobbins et al [36]	1 (5)
Ambient application	Brauner and Ziefle [32]	1 (5)
Customized DPA^a^	Paay et al [41]^b^	1 (5)
N/A^c^	Gorjani et al [35] Li et al [39] Sahu et al [43] Totty and Wade [46] Guan et al [48] Naranjo-Hernández et al [49] Demiris et al [51]	7 (35)

^a^DPA: digital personal assistant.

^b^The researchers customized a DPA to minimize participants’ previous experience and bias toward a specific device or brand.

^c^N/A: not applicable.

**Table 5:** Distribution of countries of the studies (N=20).

Country	Studies, n (%)
United States	4 (20)
United Kingdom	2 (10)
Spain	2 (10)
Saudi Arabia	2 (10)
South Korea	1 (5)
Turkey	1 (5)
Czech Republic	1 (5)
Italy	1 (5)
Denmark	1 (5)
Canada	1 (5)
Austria	1 (5)
Ireland	1 (5)
China	1 (5)
Germany	1 (5)

#### Publication Year, Country, and Type of Study

Figure 2 shows the number of studies conducted each year. The included articles were published within a 12-year period ranging from 2009 to 2020. More than half were published in 2018 and 2020 combined (11/20, 55%). The year 2018 had the highest number of publications (6/20, 30%), followed by 2019 (5/20, 25%). Only 5% (1/20) of the articles were published in 2012, 2015, 2016, and 2017, each of which represented the lowest number of articles published in a given year. Table 5 shows the distribution of studies based on country. The highest number of studies (6/20, 30%) was published in the United States, and the lowest number (1/20, 5%) was published in countries such as South Korea, Turkey, and Czech Republic. Finally, regarding the type of study, quantitative studies were the most common (15/20, 75%), followed by mixed studies (3/20, 15%) and qualitative studies (2/20, 10%).

#### Machine Learning Algorithms

Table 2 shows the machine learning algorithms used by the various studies and their corresponding authors. Overall, most studies (12/20, 60%) did not use a machine learning algorithm. K-nearest neighbor and naïve Bayes classifier were the most commonly used algorithms, with 10% (2/20) of the articles reporting each. Other algorithms such as artificial neural networks (ANNs), convolutional neural networks, and hidden Markov models were only applied once. Compared with Bayesian or neural networks, which require and use a large amount of training data sets (eg, the study by Gorjani et al [35], which used approximately 300,000 data points), support vector machine used a small number of available training sets (eg, the study by Li et al [39], which used <200 data points).

#### Goal of the Studies

Table 3 shows the overall goals of the interventions evaluated in the included studies. Activity recognition (eg, by a robot) turned out to be the most common goal (11/20, 55%), followed by activity monitoring (7/20, 35%) and biofeedback (3/20, 15%). Coaching turned out to be the least common goal, with only 5% (1/20) of the articles focusing on it.

#### Interactive Technologies

Table 4 shows the various types of technologies on different platforms implemented or evaluated in the included studies. Desktop applications (5/20, 25%) turned out to be the most common technology, followed by web applications (4/20, 20%) and mobile apps (4/20, 20%). A total of 35% (7/20) of the studies did not mention the type of technology or application evaluated.

#### Sensors

Various types of sensors were evaluated in the included articles. A total of 40% (8/20) of the studies evaluated ambient sensors [3,32,39,41,43,47,48,51]. Similarly, 40% (8/20) of the studies evaluated wearable sensors [33,35-38,45,46,49]. Both sensors turned out to be the most common in the reviewed studies, followed by robots (3/20, 15%) [34,40,44] and hybrid (ambient and wearable) sensors (1/20, 5%) [42].

#### Physical Activity

Various types of physical activity were detected, recognized, tracked, or monitored in the reviewed studies. They included ADLs, therapeutic exercise, physical exercise, and bodyweight exercise. ADLs are physical activities that individuals can perform without human assistance, including aerobic exercise, strength training, balance, dressing, toileting, ambulating, and eating. With the aid of technology, they are essential for independent living and better quality of life [53]. Therapeutic exercises are bodily movements prescribed to correct impairments and restore muscular and skeletal function and flexibility to improve strength, decrease pain, or maintain a state of well-being. They include progressive resistive exercise, balance training, strength training, and aerobic conditioning [54,55]. Physical exercises are planned, structured physical activities performed for the purpose of fitness or functional benefits. Bodyweight exercises are strength training exercises that leverage the weight of the human body as resistance (eg, push-ups and squats [56]) to enhance a range of biomotor abilities such as endurance, speed, strength, flexibility, coordination, and balance [57]. A total of 45% (9/20) of the studies [3,35,36,43,46-49,51] focused on ADLs; 30% (6/20) [34,38-40,44,45] focused on physical exercise such as running, cycling, and weight lifting; and 20% (4/20) [32,33,37,42] focused on therapeutic exercises. Only 5% (1/20) of the studies [41] focused on bodyweight exercises such as lunges, jumping jacks, and shoulder presses.

#### Study Settings

The included studies were conducted in various settings. In total, 40% (8/20) were conducted in a laboratory setting [32,35,37,39-41,48,49], 25% (5/20) were conducted in a home setting [3,32,33,43,51], and 10% (2/20) were conducted in a hospital setting [42,47]. Laboratory setting means that the study was conducted in an environment where researchers installed smart home sensors in a laboratory for data collection instead of in a real home setting, which has basic rooms and equipment for daily living. Only 5% (1/20) of the studies were conducted in a nursing home [34] and a senior residential home [47]. One-quarter of the studies (5/20, 25%) [36,38,44-46] did not specify the setting in which they were conducted.

#### Study Outcomes

We used the accuracy of the evaluated smart home technologies (eg, activity detection and recognition sensors) in the quantitative studies and the subjective judgment or conclusions of the authors in the qualitative studies to determine the effectiveness of a given technology. As shown in Table 1, a total of 75% (15/20) of the studies [3, 32-37, 39, 40, 43, 45, 46, 48, 49, 51] had a positive outcome, 10% (2/20) [38,41] had a mixed outcome, and 15% (3/20) [42,44,47] had an indeterminate outcome.

## Discussion

On the basis of the presented results, we discuss the key findings of the scoping review considering the 3 main research questions and briefly compare them with the results of previous systematic reviews.

### How Have Smart Home Technologies Been Used to Promote Physical Activity Among the General and Aging Populations?

This scoping review had a number of interesting findings based on different study characteristics. For example, the year 2018 recorded a surge in published studies on smart homes aimed at promoting physical activity (Figure 2). From 1 study per year over a 6-year period, the number of studies went from 1 in 2017 to 6 in 2018. It dipped in 2019 to 3 and increased to 5 in 2020. Overall, as of 2018, the number of published studies increased from 1 per year to at least 3 per year. This is an indication of increasing interest among researchers in smart home technologies aimed at promoting physical activity. A plausible explanation for this remarkable increase is the growing number of older people each year, especially in Western societies [58]. As shown in Table 5, three-quarters of the studies (15/20, 75%) were conducted in Western countries, with the United States, United Kingdom, and Spain accounting for half (10/20, 50%). For the growing number of older people to function independently and have active lives, there is a need to leverage smart home technologies that promote physical activity to support aging in place [53].

Another key finding is that there was an equal split between ambient and wearable technologies, each of which accounted for 40% (8/20) of the studies. Among the ambient technologies was the noncontact sensor (Doppler radar–based system) presented by Li et al [39]. The system was able to recognize respiration, body movement, and physical activity. This system was introduced as a novel solution for multiple health care applications and complemented traditional smart home sensor systems for assisted living. Particularly, Cahill et al [47] recommended that residential care facilities provide a compassionate, social, and ethically oriented user experience for both patients and caregivers. Stating that smart home technology goes beyond electronic devices and sensors, Cahill et al [47] proposed 3 requirements for new AAL technology that should be considered to improve social relationships, well-being, quality of care, and independence [53]. They include the state of the user (health and wellness), the state of the home or environment, and the state of care delivery (eg, ADL support and level of care contact). They advocated that future AAL technology should be predicated on the “biopsychosocial models of wellness, concepts of home and support relationships between older adults and members of the personal and professional community” [47]. The biopsychosocial model holds that biological, psychological (eg, thoughts, emotions, and behaviors), and social (eg, socioeconomical, socioenvironmental, and cultural) factors play a substantial role in health and disease. In other words, it states that health and disease can be best understood in terms of the interaction of biological, psychological, and social factors rather than solely in biological terms proposed by the classic biomedical model [59,60].

Furthermore, 15% (3/20) of the studies [34,40,44] were based on robot technologies with multiple features, including learning, demonstrating, monitoring, recommending exercises, and providing feedback. For example, Costa et al [40] presented a physical assistant robot system (PHAROS) to promote and aid older adults in their daily physical activities at home. With the aid of a camera, PHAROS, which recommended and monitored exercises, used novel deep learning methods (such as recurrent neural networks) to accurately identify the body motions and positions of the user. This enabled it to intelligently guide the user (an older person) to perform a physical exercise safely and successfully.

Recognition of activities turned out to be the most frequent goal of the experimental studies (Table 3), with 55% (11/20) focusing on it. For example, Gorjani et al [35] investigated the application of ANNs and logistic regression to recognize the activities performed by smart home occupants. Following in second place was monitoring of activities, with 35% (7/20) of the studies investigating it. For example, Yoh et al [52] evaluated a cardiac rehabilitation (CR) exercise smartphone app connected to a wearable device worn on the wrist. The smartphone app enabled the patient to record and monitor their exercise. With the aid of a medical station, a CR specialist was able to review and monitor the patient’s exercise records and prescribe new types of CR exercises, which were sent to the patient via the smartphone app. The wearable device measured vital signs such as instantaneous heart rate and informed the patient about the exercise stage (eg, resting, walking, and running) they were currently in. In addition, the device automatically recommended appropriate exercise intensity in real time with the aid of light-emitting diodes. More importantly, some of the smart home technologies had 2 or more goals. A case in point is the exercise robot evaluated by Görer et al [34], which learned body movements, mimicked them through demonstration, monitored exercise, and provided feedback to the user.

ADLs turned out to be the most frequently studied activities of interest, with nearly half (n=9, 45%) of the 20 studies focusing on them. Some of the ADLs included mobility, eating, toileting, and physical exercise, to mention but a few [47]. Totty and Wade [46], for example, investigated the feasibility of classifying ADLs into 4 functional categories among 10 healthy adults without disabilities using a noninvasive sensor and a k-nearest neighbor machine learning algorithm. The 4 categories were *nonfunctional* (eg, arm sway during walking), *non–task-related* (eg, holding an object), task-related (moving an object), and high *exertion* (eg, scrubbing). They found this approach useful for quantifying ADLs in ambient settings and as a more ecologically valid measure of function among healthy and nonhealthy people. Second to ADLs was physical activity, with 25% (5/20) of the studies evaluating it. For example, Li et al [39] and Syed et al [45] focused on the recognition of physical activity such as cycling, walking, running, jumping, standing, sitting, and turning. Moreover, few studies (6/20, 30%) specifically focused on therapeutic exercises [32,33,37,42] and bodyweight exercises [38,41], which can also be considered physical activity.

Desktop applications turned out to be the most evaluated interactive smart home technology, with 25% (5/20) of the included studies focusing on it. In second place were web applications and mobile apps, each of which was evaluated in 20% (4/20) of the included studies (Table 4). Regarding desktop applications and robots, for example, Schmied et al [44] evaluated digital health coaches in transitioning into retirement. Apart from promoting physical activity, the coach (a robot or avatar) provided cognitive, emotional, and social support [44]. The authors found that, to increase acceptance, coaches should provide users with autonomy, such as being able to control the coach, including turning it on and off at any given time. Moreover, the participants requested data security, availability of the coach on different devices (eg, smartphones and tablets), humanlike attributes and interactions, and personalization of the coach [44].

It is noteworthy that robots are becoming popular in supporting the growing aging population to age in place [61]. Research shows that, by 2031, nearly twice as many older adults will require care. Robots will help bridge this gap as the older adult population outpaces the growth of long-term care workers in the industry. As Dr Nejat puts it, “We’re about five years away from seeing robots more commonly used in the home or at [seniors’] residences” [62]. Although some have argued that robots are still not set for widespread commercialization, partly because of “high cost,” others have argued that, just as we are seeing more people use smart home systems such as Amazon Alexa and Google Home, we will see the same happen with robotics in a few years to come. However, despite the bright prospects of robots, it remains unclear whether people would embrace them given their mechanical characteristics, which may be a far cry from human characteristics at this stage of their development. With that said, the closer robots mimic human characteristics such as facial expressions, body gestures, and emotional intelligence, the more likely they are to be accepted by their intended users. For example, in the study by Schmied et al [44], some of the participants admitted that they did not like the appearance of the robot, whereas others found it “cute.” The general feeling about “the robot was that it looked too humanoid” [44]. In the meantime, the more closely robots mimic smart home products such as the Amazon Alexa, Google Nest Hub, and the iRobot vacuum cleaner robot [61], which people are already familiar with, and the more affordable they are, the more likely they are to be accepted by the target users [62]. However, there is a need for more research on the acceptance and effectiveness of robots (and other smart home technologies) among the aging population in home settings, where they will end up eventually supporting older people to age in place and function independently.

### How Effective Are Smart Home Technologies for Promoting Physical Activity Among the General and Aging Populations?

At least 40% of the smart home technologies used machine learning algorithms to detect, recognize, or classify ADLs, including physical exercises. For example, the integrated system by Syed et al [45] (comprising Internet of Medical Things and wearable devices), which used the naïve Bayes classifier, was able to recognize 12 physical activities with an overall accuracy of 97.1%. The author considered this system an optimal solution for recognizing the physical activities of older people remotely monitored [19]. Similarly, the PHAROS by Costa et al [40] used novel deep learning methods (such as recurrent neural networks and convolutional neural networks) to detect and classify physical exercises via movements and positions with 97.35% accuracy. Moreover, the passive radar system by Li et al [39] for monitoring exercise, which used support vector machine, had an accuracy ranging from 85% to 98.65%, whereas the activity recognition system by Gorjani et al [35], which used ANNs, had an accuracy ranging from 91.2% to 100%. However, the study by Naranjo-Hernández et al [49] on using a smart wearable sensor for distinguishing and classifying ADLs (including physical activity) did not report the accuracy rate. Overall, the accuracy of most of the machine learning algorithms was ≥90%. More importantly, most studies (13/20, 65%) had a positive outcome, and 15% (3/20) had a mixed (positive as well as negative) outcome. Altogether, smart home technologies have the potential to promote the physical activity of the target users.

### What Research Gaps Need to Be Filled and New Research Opportunities Need to Be Explored?

We presented a scoping review of the extant work on smart home technologies aimed at promoting physical activity among the general population and fostering functionally independent living among the aging population. Although we uncovered some interesting findings, such as the increasing interest in this line of research among scholars, concentration of research in Western societies, greater focus on the younger population, and use of robotics, many gaps remain to be filled, which present new research opportunities.

First, as shown in Table 1, most of the interventions (7/20, 35%) [32-34,39,41,46,49] focused on the younger population (aged 15-47 years). This may be because this age group is the most digitally literate and most likely to engage in physical activity. As such, researchers might have found it more convenient to investigate this population regarding the use of smart home technologies to promote physical activity. Only 20% (4/20) of the studies [32,34,37,50] focused on the older adult population (aged ≥64 years), and 15% (3/20) [32,34,41] on the middle-aged population (aged 48-63 years). Over half of the studies (11/20, 55%) did not report the ages of the study participants. These findings are an indication that there is a scarcity of studies on the aging population, especially the older adult population aged ≥64 years, who are most likely to rely on smart home technologies for promoting physical activity and aging in place. Thus, there is a need for more studies in this area as the population aged ≥64 years increases year by year, especially in Western countries [58]. The results and recommendations of studies focusing on the aging population will provide beneficial insights into the potential of smart home technologies in promoting physical activity, especially ADLs, and fostering independent living.

Second, we found shortcomings in the research design and the reporting of participants’ demographic information. For example, we found that 90% (18/20) of the studies lacked a control group, as evidenced in Table 1. In experimental research, control groups are necessary to determine the effectiveness of a given intervention. Hence, we recommend that future work on using smart home technologies to promote physical activity integrate control groups into the research design. The inclusion of a control group in the research design will allow researchers to compare the experimental and control groups to determine the effectiveness of the intervention. Moreover, over two-thirds of the studies (11/20, 55%) provided insufficient demographic information about the participants under investigation. For example, 25% (5/20) of the studies [40,42,43,45,47] provided no information on the health condition, gender, or age of the participants.

Third, only 25% (5/20) of the reviewed studies [32,34,41,44,47] focused on technology acceptance or the ethical considerations that underscore the design of smart home technologies aimed at promoting independent living through physical activity [19]. Given the increasing aging population, some of whom may not be digitally literate, there is a need to study the acceptance of smart home technologies for promoting physical activity, including ADLs [6]. Despite recent research showing that an increasing number of older people are embracing digital lives, they face unique barriers because of age-related challenges, which may make it difficult for them to embrace smart home technologies aimed at promoting physical activity and independent living [63]. Although smart home technology adopters had less overall concern and a higher level of perceived data protection toward digital assistants than nonadopters [64], it remains to be seen how this finding varies across different demographic groups based on country, culture, age, sex, and gender. Hence, we recommend more research in this area.

Fourth, as shown in Table 3, we found that most of the studies in the review focused on activity recognition (11/20, 55%) [3,35,36,38-40,42,45,46,48,49] and activity monitoring (7/20, 35%) [33,34,38,40,45,47,51] and were conducted in a laboratory setting. These findings suggest the need to expand future research beyond activity recognition and monitoring and the laboratory setting. Laboratory settings have restrictions as they are not representative enough of real-life scenarios because of the control of several variables present in actual environments. This reduces the external validity of such research as it is often hard to migrate experimental settings (test conditions and procedures) to real-life situations [65]. Hence, there is a need for more research in real-life settings such as homes, nursing homes, and senior residential homes. We also found that most studies (10/20, 50%) focused on desktop applications [3,32,38,40,44], web applications [33,40,42,47], and mobile apps [33,37,45,47]. As shown in Table 4, there is a scarcity of research on smartwatch apps and ambient applications. Hence, we recommend that more research be conducted in these areas in the future.

Fifth, few studies (6/20, 30%) presented evidence on the effect of smart home technologies on physical exercise performance, with only 5% (1/20) evaluating the effect of incorporating persuasive design into exercise-promoting smart home technologies. Paay et al [41] investigated the persuasive capability of a digital personal assistant aimed at promoting bodyweight exercises. They assessed the effectiveness of 3 types of persuasive feedback (suggestion, virtual reward, and praise) compared with the no-feedback condition. They found that all 3 types of feedback had a positive effect on motivating participants to continue exercising and putting effort into it. Only 10% (2/20) of the included studies [32,44] addressed the perception and acceptance of smart home technologies that promote physical activity, with one of them investigating the effect of age. Brauner and Ziefle [32] found that age was negatively associated with physical activity performance, perceived usefulness, ease of use, and overall acceptance. Hence, there is a need for future work, especially randomized controlled trials in home or residential settings, that aims to investigate the effectiveness and acceptance of exercise-promoting smart home technologies. In particular, there is a need for cross-country, cross-cultural, or cross–group research to understand the generalizability of findings from one national population or group to another or uncover their differences. Particularly in a smart home context, there is a need to compare the effectiveness of different machine learning algorithms aimed at recognizing, detecting, or classifying physical activities and how the level of effectiveness affects the adoption and use of the technologies.

More importantly, as robots set to “invade” our homes in the next few years, especially because of the growing aging population and the shortage of long-term care workers in Western societies [61,62], there is a need to study their design as persuasive tools and social actors that have the potential to influence human thinking and behaviors. Designing them as ethically responsible and personalizable persuasive technologies, for example, can make them more appealing and increase adoption, use, and the user experience [66]. This may entail equipping them with different human characteristics and interactions, such as facial expressions, body gestures, emotional intelligence, and affective qualities, to mention but a few, and investigating how each type of human characteristic and interaction affects users’ acceptance, use, and the user experience. This, in addition to making them affordable, has the potential to increase robot adoption in the near future [61,62,66].

### Comparison With Other Review Results

We compared the objectives and results of our scoping review with those of similar systematic reviews that we found in our search before conducting the review and after submitting the preprint. Previous and more recent systematic reviews have focused on smart home technologies in general [30,67], monitoring and improving the health outcomes of the older population aged ≥64 years [11,19] or older people with certain health conditions [24,25,68]. Hence, unlike the systematic reviews (presented in the *Introduction* section) that focused mainly on the older population and those with chronic conditions, our scoping review focused on the general population with and without health conditions. For example, Facchinetti et al [24], Liu et al [25], and Lussier et al [26] demonstrated that smart home technologies hold great potential in promoting positive health outcomes (such as physical functioning and management of chronic diseases and depression) among the older adult population, while we found that they hold promise in promoting physical activity among the young population. More importantly, our review revealed several research gaps in the use of smart home technologies to promote physical activity. The research gaps include limited work in non-Western regions such as Africa and South America, among the older adult population, among those with chronic conditions, on the use of persuasive design, in real-world settings, and using control groups. For example, in our review, we only found 10% (2/20) of studies that focused on the older adult population with chronic diseases. They included the studies by Fung et al [37] and Skubic et al [50] that focused on older people with Parkinson disease and chronic illnesses, respectively. This is an indication that there is a need for further studies to examine the effectiveness and adoption of smart home technologies for supporting physical activity among people with chronic conditions.

### Limitations

This scoping review has a number of limitations. The first limitation concerns database search decisions. For example, our decision to limit our database search to articles published in English might have made us miss relevant studies published in other international languages such as French and German. Similarly, our decision to search only 6 databases might have led us to miss relevant articles that were indexed in other databases. The second limitation is that, while screening, there is the possibility that we screened out some relevant articles owing to human error or incorrect subjective judgment. The third limitation is that, between when the included articles were retrieved from the databases and the final publication of the scoping review, more studies not included in the scoping review may have been published. Hence, the scoping review may not fully represent all the published articles on this topic to date. The fourth limitation concerns the elicitation of data from the included articles. Given that we used subjective judgment, the elicitation of the charted data might have been prone to human error, including misinterpretation or misclassification of elicited data into thematic categories. Future reviews should aim to bridge these gaps.

### Conclusions

Smart home technologies are becoming more and more popular, especially for health care service delivery [6,67]. Research on leveraging these technologies to promote physical activity in laboratory and home settings is gaining traction in the research community, especially involving robots aimed at promoting exercise and aging in place [61,62]. Hence, there is a need to gain insights into how smart home technologies have been used to support and promote the physical activity required by the aging population and people with certain health conditions to live and function independently. This scoping review contributes to such insights by surveying the landscape, synthesizing emerging themes, and reporting the findings. The key findings include (1) smart home technologies have the potential to improve physical activity among the young population (aged 15-47 years), (2) most of the research on smart home technologies for promoting physical activity is concentrated in the laboratory setting, and (3) most of the smart home technologies are focused on activity recognition and monitoring. This scoping review serves as a first step in determining the value and need to carry out a full systematic review of smart home technologies for promoting physical activity in the near future. As the years go by, we look forward to seeing more published studies on the subject, as evident in Figure 2, in which there is an increase in the number of studies after 2017, with the number per year ranging from 3 to 6.

## Appendix

Multimedia Appendix 1 Detailed search strings for each electronic database.

Multimedia Appendix 2 PRISMA-ScR (Preferred Reporting Items for Systematic Reviews and Meta-Analyses extension for Scoping Reviews) checklist.

## Abbreviations

AAL: Active Assisted Living
ADL: activity of daily living
ANN: artificial neural network
CR: cardiac rehabilitation
IoT: Internet of Things
PHAROS: physical assistant robot system
PRISMA-ScR: Preferred Reporting Items for Systematic Reviews and Meta-Analyses extension for Scoping Reviews

## Data Availability

All data generated or analyzed during this study are included in this paper.
