# Supplementary material for: Using Smart Home Technologies to Promote Physical Activity Among the General and Aging Populations: Scoping Review
Source: J Med Internet Res. 2023 May 12;25:e41942. doi: 10.2196/41942 (PMC10221512; doi:10.2196/41942)
Supplement: Multimedia Appendix 1 [file jmir_v25i1e41942_app1.docx]

**Appendix A: Detailed Search Strings for Each Electronic Database.**

| **Databases** | **Search Strings** |
| --- | --- |
| Pubmed | #1: smart home*[tiab] OR smarthome*[tiab] OR Active Assisted Living[tiab] OR Ambient Assisted Living[tiab]  #2: exercise*[tiab] OR fitness[tiab] OR physical activit*[tiab] OR activit* monitor*[tiab]  #3: #1 AND #2 |
| CINAHL | #1: TI (“smart home*” OR smarthome* OR “Active Assisted Living” OR “Ambient Assisted Living”)  #2: TI (exercise* OR fitness OR “physical activit*” OR “activit* monitor*”)  #3: #1 AND #2  #1: AB (“smart home*” OR smarthome* OR “Active Assisted Living” OR “Ambient Assisted Living”)  #2: AB (exercise* OR fitness OR “physical activit*” OR “activit* monitor*”)  #3: #1 AND #2 |
| Scopus | (TITLE-ABS-KEY ( "smart home*" OR smarthome* OR "Active Assisted Living" OR "Ambient Assisted Living" )  AND  TITLE-ABS-KEY (exercise* OR fitness OR "physical activit*" OR "activit* monitor*")) |
| IEEE Xplore | ("Publication Title": "smart home*" OR "Publication Title": smarthome* OR "Publication Title": "Active Assisted Living" OR "Publication Title": "Ambient Assisted Living")  AND  ("Publication Title": exercise* OR "Publication Title": fitness OR "Publication Title": "physical activit*" OR "Publication Title": "activit* monitor*")  ("Abstract": "smart home*" OR "Abstract": smarthome* OR "Abstract": "Active Assisted Living" OR "Abstract": "Ambient Assisted Living")  AND  ("Abstract": exercise* OR "Abstract": fitness OR "Abstract": "physical activit*" OR "Abstract": "activit* monitor*")  ("Author Keywords": "smart home*" OR "Author Keywords": smarthome* OR "Author Keywords": "Active Assisted Living" OR "Author Keywords": "Ambient Assisted Living")  AND  ("Author Keywords": exercise* OR "Author Keywords": fitness OR "Author Keywords": "physical activit*" OR "Author Keywords": "activit* monitor*") |
| ACM Digital Library | [[Title: "smart home*"] OR [Title: smarthome*] OR [Title: "active assisted living"] OR [Title: "ambient assisted living"]]  AND  [[Title: exercise*] OR [Title: fitness] OR [Title: "physical activit*"] OR [Title: "activit* monitor*"]]  [[Abstract: "smart home*"] OR [Abstract: smarthome*] OR [Abstract: "active assisted living"] OR [Abstract: "ambient assisted living"]]  AND  [[Abstract: exercise*] OR [Abstract: fitness] OR [Abstract: "physical activit*"] OR [Abstract: "activit* monitor*"]]  [[Keywords: "smart home*"] OR [Keywords: smarthome*] OR [Keywords: "active assisted living"] OR [Keywords: "ambient assisted living"]]  AND  [[Keywords: exercise*] OR [Keywords: fitness] OR [Keywords: "physical activit*"] OR [Keywords: "activit* monitor*"]] |
| Web of Science | "smart home*" OR smarthome* OR "Active Assisted Living" OR "Ambient Assisted Living" (Title)  AND  exercise* OR fitness OR "physical activit*" OR "activit* monitor*" (Title)  "smart home*" OR smarthome* OR "Active Assisted Living" OR "Ambient Assisted Living" (Abstract)  AND  exercise* OR fitness OR "physical activit*" OR "activit* monitor*"(Abstract)  "smart home*" OR smarthome* OR "Active Assisted Living" OR "Ambient Assisted Living" (Author Keywords)  AND  exercise* OR fitness OR "physical activit*" OR "activit* monitor*"(Author Keywords) |
